# Whole-Genome Sequencing Reveals Virulence and Antimicrobial Resistance Determinants of *Lactococcus garvieae* Causing Lactococcosis in Cage-Cultured Nile Tilapia (*Oreochromis niloticus*) in Thailand

**DOI:** 10.3390/ijms27177582

**Published:** 2026-08-24

**Authors:** Putita Chokmangmeepisarn, Yosapon Adisornprasert, Pakapon Meachasompop, Benchawan Kumwan, Pimrawee Chaemlek, Prapansak Srisapoome, Kednapat Sriphairoj, Sittichai Hatachote, Niyada Umputhorn, Chonthicha Choppradit, Pichasit Sangmek, Channarong Rodkhum, Anurak Uchuwittayakul

**Affiliations:** 1 Department of Microbiology, Faculty of Science, Kasetsart University, Bangkok 10900, Thailand; 2 Special Research Incubator Unit for Development and Application of Vaccine Delivery Systems for Aquatic Animals, Department of Aquaculture, Faculty of Fisheries, Kasetsart University, Bangkok 10900, Thailand; yosapon.ad@ku.th (Y.A.); pakapon.meac@ku.th (P.M.); benchawan.kumw@ku.th (B.K.); pimrawee.ch@ku.th (P.C.); ffispssp@ku.ac.th (P.S.); 3 Center of Excellence in Aquatic Animal Health Management, Faculty of Fisheries, Kasetsart University, Bangkok 10900, Thailand; 4 Laboratory of Aquatic Animal Health Management, Department of Aquaculture, Faculty of Fisheries, Kasetsart University, Bangkok 10900, Thailand; 5 Faculty of Natural Resources and Agro-Industry, Kasetsart University, Chalermphrakiat Sakon Nakhon Province Campus, Sakon Nakhon 47000, Thailand; kednapat.sr@ku.th (K.S.); sittichai.h@ku.th (S.H.); niyadaum@gmail.com (N.U.); chonticha.020428@gmail.com (C.C.); pichasit.sa@ku.th (P.S.); 6 Center of Excellence in Fish Infectious Diseases (CE FID), Faculty of Veterinary Science, Chulalongkorn University, Bangkok 10330, Thailand; channarong.r@chula.ac.th

**Keywords:** *Lactococcus garvieae*, lactococcosis, Nile tilapia, whole-genome sequencing, virulence factors, antimicrobial resistance

## Abstract

Lactococcosis is an important bacterial disease affecting farmed fish worldwide and is primarily associated with *Lactococcus garvieae*, *Lactococcus petauri*, and *Lactococcus formosensis*. In Thailand, information on *L. garvieae* infection in tilapia remains limited, particularly regarding genome-based identification, virulence determinants, and antimicrobial resistance profiles. This study characterized two *L. garvieae* isolates, AAHM-LG2501 and AAHM-LG2509, recovered from a lactococcosis outbreak in cage-cultured Nile tilapia (*Oreochromis niloticus*) in Ubon Ratchathani province, Thailand. Both isolates exhibited typical phenotypic characteristics of *L. garvieae*, including Gram-positive cocci, alpha hemolysis, positive capsule staining, and positive carbohydrate fermentation. Whole-genome sequencing confirmed both isolates as *L. garvieae*, with genome sizes of approximately 1.95 Mb and a G + C content of 38.9%. Genome-based taxonomic analysis supported species identification based on dDDH and ANI values, and both isolates were assigned to sequence type ST95 and serotype I. Virulence factor analysis identified 288 virulence-associated genes representing 97 virulence factors across 14 functional categories. Capsule-associated genes were prominent, together with genes involved in heme uptake, adhesion, hemolysis, stress survival, biofilm formation, and host adaptation. Ten capsule biosynthesis genes, including *cpsABCFGKO*, *cps4A*, and *cps4I*, as well as LPxTG cell wall anchor protein genes, were detected. Antimicrobial susceptibility testing showed resistance to nalidixic acid, oxolinic acid, and oxacillin, while reduced inhibition zones were observed for enrofloxacin and sulfamethoxazole-trimethoprim. Genome analysis identified predicted antimicrobial resistance determinants, including *lsaD*, *vanT*, *vanY*, and *mdtA*. Resistance-associated protein variants were detected in *gyrA* and *gyrB*, suggesting that target alteration may contribute to fluoroquinolone resistance. Overall, this study provides genome-level evidence of virulence and antimicrobial resistance determinants in *L. garvieae* from Thai tilapia and highlights the importance of whole-genome sequencing for accurate diagnosis, epidemiological surveillance, and disease management in aquaculture.

## 1. Introduction

Lactococcosis has emerged as a significant bacterial disease threatening the aquaculture industry and affecting a wide range of cultured aquatic species. Three species, including *Lactococcus garvieae*, *Lactococcus petauri*, and *Lactococcus formosensis*, have been known to cause lactococcosis in fish [1]. Among the recognized causative agents of lactococcosis, *L. garvieae* is frequently associated with peracute to acute hemorrhagic septicemia in fish, leading to significant morbidity and mortality in both freshwater and marine aquaculture systems [2,3,4,5,6,7,8]. Affected fish typically exhibit clinical signs similar to streptococcosis such as exophthalmia, ocular opacity, skin hemorrhage, darkening of the skin, erratic swimming, and ascites [2,4,7]. Given that lactococcosis outbreaks are typically associated with warm-water conditions and are most frequently reported during the summer season, climate change and rising water temperatures may contribute to the increasing occurrence and geographic expansion of the disease [9].

In Thailand, *L. garvieae* mainly affects cultured Nile tilapia (*Oreochromis niloticus*) and red tilapia (*Oreochromis* sp.), causing recurrent disease outbreaks with cumulative mortality rates ranging from 2% to 24% in natural infection [2,8,10]. Experimental infection studies have revealed substantial strain-dependent and dose-dependent variation in the virulence of *L. garvieae*, resulting in cumulative mortality rates ranging from 0% to 100% [2,8]. This variability in pathogenicity has been associated with multiple virulence determinants, including capsule formation, hemolysins, and adhesins, which are believed to play important roles in host colonization, immune evasion, and disease development [11,12,13,14]. The capsule-associated gene cluster (CGC), which comprises genes involved in exopolysaccharide (EPS) biosynthesis and capsule formation, has been identified in highly virulent *L. garvieae* isolates and disruption of conserved genes within the CGC resulted in a marked reduction in virulence [12,14].

Antibiotic treatment is commonly employed to control lactococcosis outbreaks in aquaculture; however, the extensive use of antimicrobial agents may contribute to the emergence and spread of antimicrobial resistance. Several *L. garvieae* isolates have been reported to exhibit resistance to multiple classes of antibiotics, including sulfonamides, fluoroquinolones, beta-lactams, macrolides, and lincosamides [4,5,11,14,15,16]. Antimicrobial resistance (AMR) genes such as *lsaD*, *mdtA*, and *ermB* have been identified in the majority of *L. garvieae* isolates examined across multiple studies [5,11,14]. In addition, plasmid-borne tetracycline resistance genes, including *tetL* and *tetS*, have also been detected in some isolates, highlighting the potential for horizontal dissemination of antimicrobial resistance determinants [1,11,17].

Whole-genome sequencing (WGS) and comparative genomic analyses have become powerful tools for the characterization of bacterial pathogens. In addition to enabling comprehensive identification of virulence- and antimicrobial resistance-associated determinants, WGS provides high-resolution species-level identification and can accurately differentiate closely related species. This is particularly important for fish-associated *Lactococcus* species, including *L. garvieae*, *L. petauri*, and *L. formosensis*, which share similar phenotypic characteristics, clinical manifestations, and biochemical profiles, often leading to misidentification by conventional methods. Therefore, the present study aimed to characterize *L. garvieae* isolates recovered from a disease outbreak in cage-cultured Nile tilapia in Thailand using whole-genome sequencing focusing on virulence-associated genes and AMR determinants, together with phenotypic characterization and antimicrobial susceptibility testing.

## 2. Results

### 2.1. Bacterial Isolates and Morphological Characteristics

**Figure 1:**
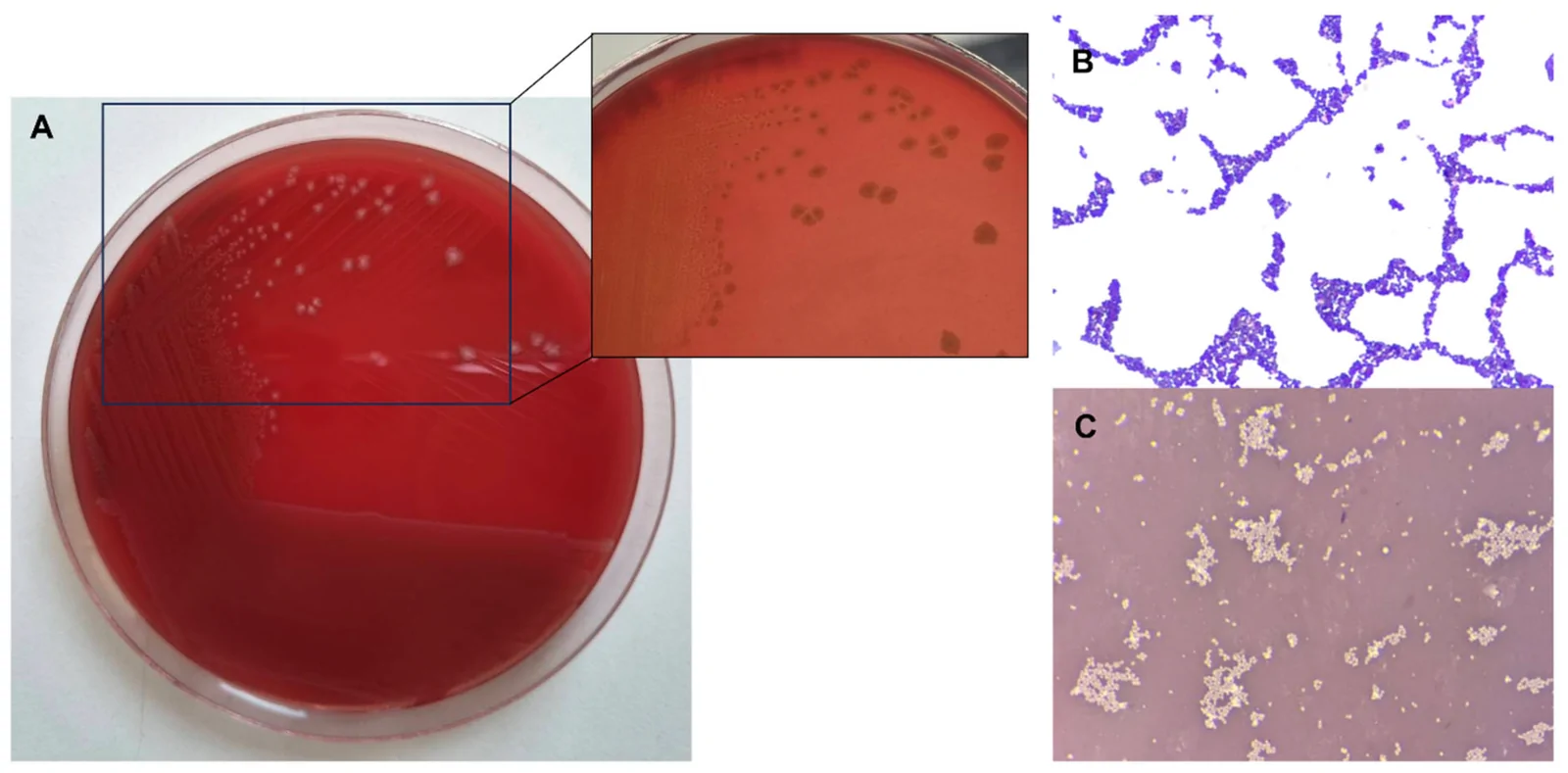
Phenotypic characteristics of *L. garvieae* AAHM-LG2501 and AAHM-LG2509. (**A**) Colony morphology and hemolysis appearance on blood agar; (**B**) bacterial cell morphology with Gram staining; (**C**) capsule staining under light microscope. Both AAHM-LG2501 and AAHM-LG2509 showed identical morphological characteristics.

Both *L. garvieae* isolates exhibited small round opaque white to cream colonies with alpha hemolysis on blood agar (Figure 1A). Gram staining revealed Gram-positive cocci with diplococci and short chain cocci (Figure 1B). The results of biochemical tests revealed that both isolates were negative to catalase, oxidase, motile, indole, and MR tests while positive to the VP test (Table 1). Moreover, both isolates exhibited positive results against all carbohydrate fermentation tests. For decarboxylase tests, all isolates were positive to arginine and negative to ornithine and lysine. Capsule staining revealed the presence of a capsule surrounding the bacterial cells (Figure 1C).

### 2.2. Genome Features

**Figure 2:**
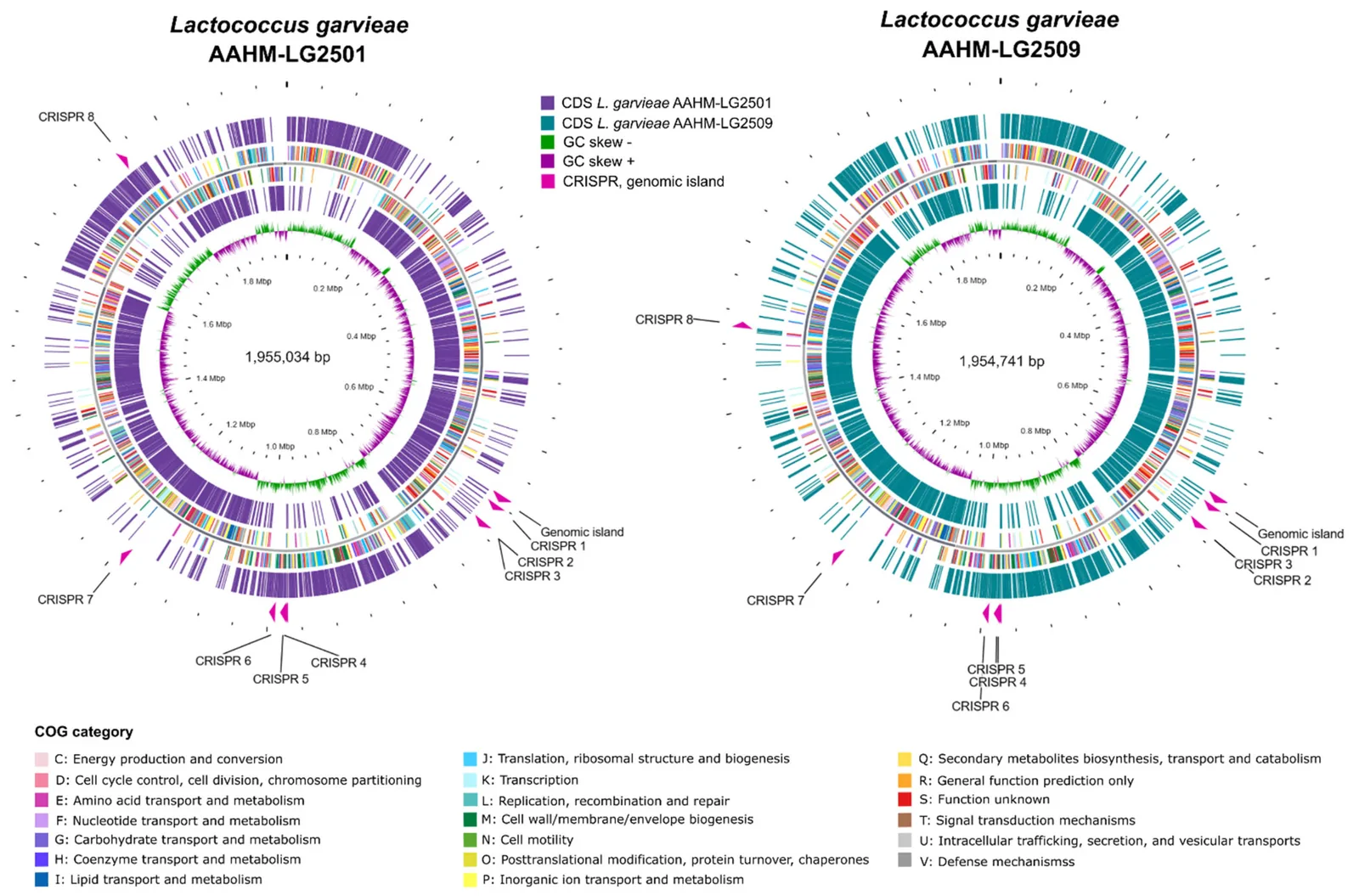
Circular genome maps of *L. garvieae* AAHM-LG2501 and AAHM-LG2509. The concentric rings display the following features, from the outermost to innermost ring: (1) CRISPR loci and genomic island, with pink arrows indicating the location and direction of the reading frame; (2) forward strand CDS; (3) COG functional categories for forward strand CDS; (4) contig backbone; (5) COG functional categories for reverse strand CDS; (6) reverse strand CDS; and (7) GC skew, where green indicates a positive skew and purple indicates a negative skew.

The genome sizes of *L. garvieae* AAHM-LG2501 and AAHM-LG2509 were 1,955,034 bp and 1,954,741 bp, respectively, with 38.9% G + C content. The genome sequences were deposited to the NCBI database under BioProject: PRJNA1455887 with accession numbers JCAFHE000000000 (AAHM-LG2501) and JCAFHD000000000 (AAHM-LG2509). The *L. garvieae* AAHM-LG2501 genome encodes for 1866 genes, 7 rRNA, 50 tRNA, 8 CRISPR loci, and one genomic island, whereas the *L. garvieae* AAHM-LG2509 genome encodes for 1870 genes, 7 rRNA, 50 tRNA, 8 CRISPR loci, and one genomic island (Figure 2).

The numbers of genes that were functionally annotated from all databases of both *L. garvieae* AAHM-LG2501 and AAHM- LG2509 were identical except the NCBI nr (Table 2). GO, eggNOG, and KEGG databases identified genes ranging from 1845–1847 genes across total CDS while 288 and 171 genes were detected by VFDB and CARD, respectively.

Annotation against the NCBI nr database revealed that the majority of predicted genes from both isolates were assigned to *L. garvieae* (85.4%) followed by *Lactococcus* sp. (12.9%) and other Lactococcus species including *Lactococcus lactis* (0.3%), *L. petauri* (0.3%), and *Lactococcus piscium* (0.1%) (Supplementary Table S1).

### 2.3. Genome-Based Identification and Phylogeny

**Figure 3:**
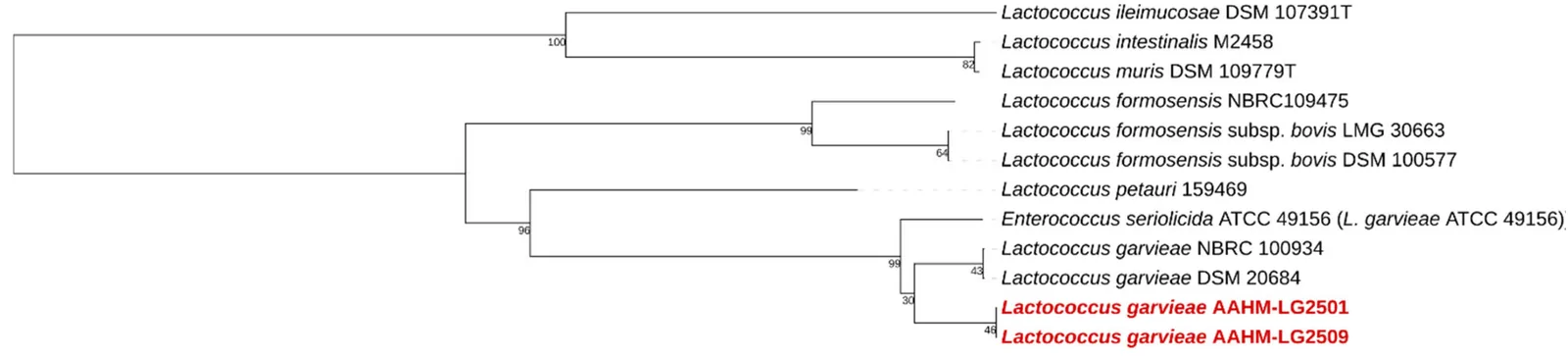
Phylogenomic tree showing relationship between *L. garvieae* of this study (red color) and type strains of *Lactococcus* spp. The tree was inferred based on the GBDP distance formula d5 and rooted at the midpoint. Number at nodes represent GBDP pseudo-bootstrap support values from 100 replicates. Scale bar represents substitutions per nucleotide position.

The GBDP-based phylogenetic tree indicated phylogenetic relationship between the two *L. garvieae* isolates and the reference strain (Figure 3). The *L. garvieae* AAHM-LG2501 and AAHM-LG2509 are classified into the same clade with type strain *L. garvieae* NBRC 100943, *L. garvieae* DSM 20684, and *L. garvieae* ATCC 49156 (formerly *Enterococcus seriolicida*).

**Figure 4:**
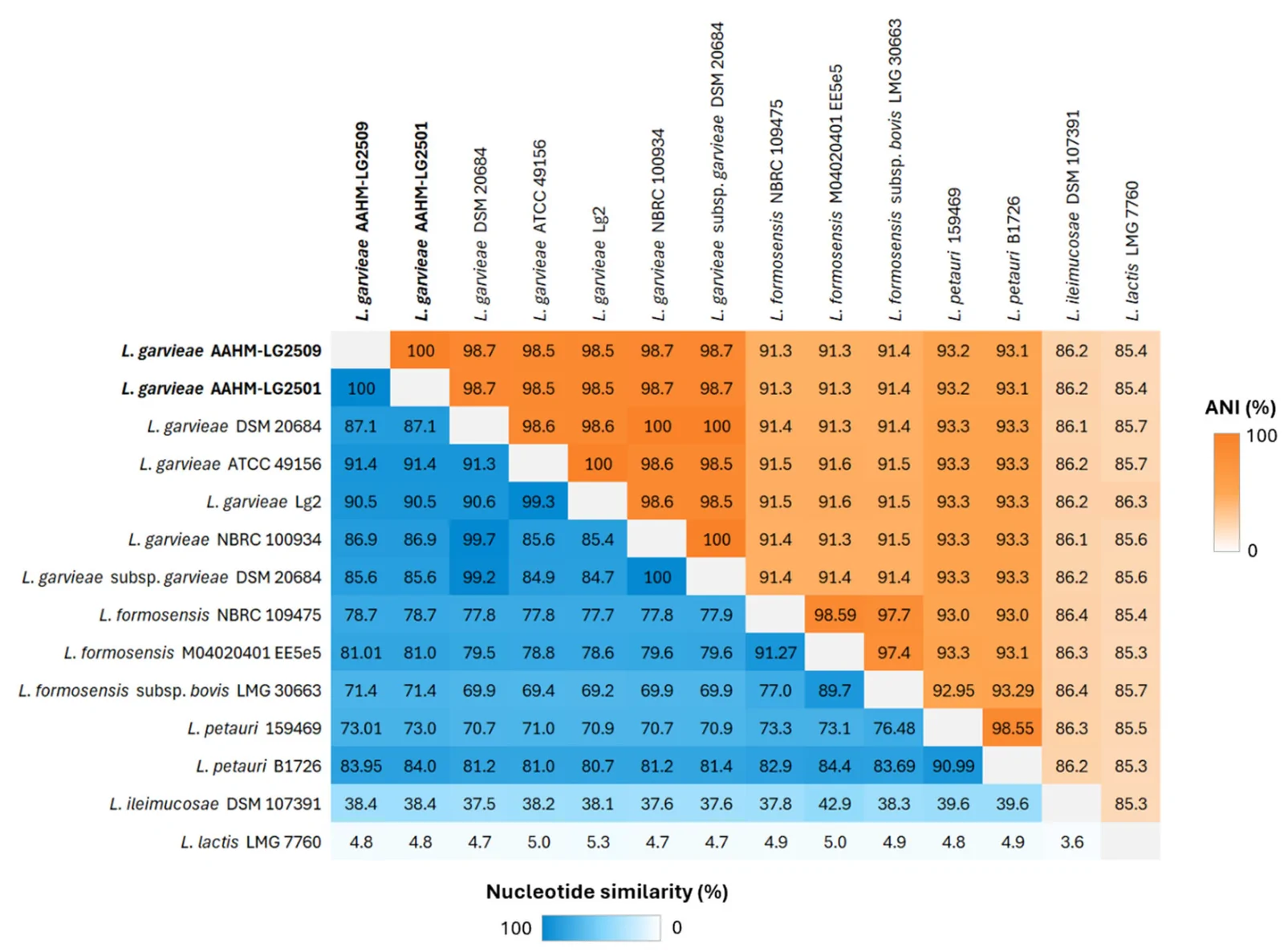
Heat map showing percent average nucleotide identity (ANI) (upper triangle) and percent nucleotide similarity (lower triangle) of *L. garvieae* of this study (bold) and other *Lactococcus* spp. Color gradient bar represents percentage of ANI (orange) and nucleotide similarity (blue).

Taxonomic thresholds including dDDH and ANI confirmed that *L. garvieae* AAHM-LG2501 and AAHM-LG2509 belong to *L. garvieae* with values exceeding species delineation thresholds (dDDH > 70%, ANI > 95%). The *L. garvieae* of this study exhibited the highest dDDH and ANI values against *L. garvieae* and *E. seriolicida* genomes with 88.6–84.4% and 98.5–98.7%, respectively (Figure 4, Table 3). The G + C difference between the *L. garvieae* of this study and type strains showing the lowest G + C difference percentage among *L. garvieae* and *L. ileimucosae* type strains ranged from 0.04–0.34%, with *L. garvieae* ATCC 49156 possessing the lowest value. The MLST scheme used to assign the sequence type (ST) of *L. garvieae* was based on *als*, *atpA*, *galP*, *gapC*, *gyrB*, *rpoC*, and *tuf*. The results showed that both *L. garvieae* AAHM-LG2501 and AAHM-LG2509 belong to ST95. Both *L. garvieae* AAHM-LG2501 and AAHM-LG2509 were assigned to serotype I based on in silico serotyping using serotype-specific PCR primers with predicted amplification products of 285 bp.

### 2.4. Functional Annotation and Virulence Factors

**Figure 5:**
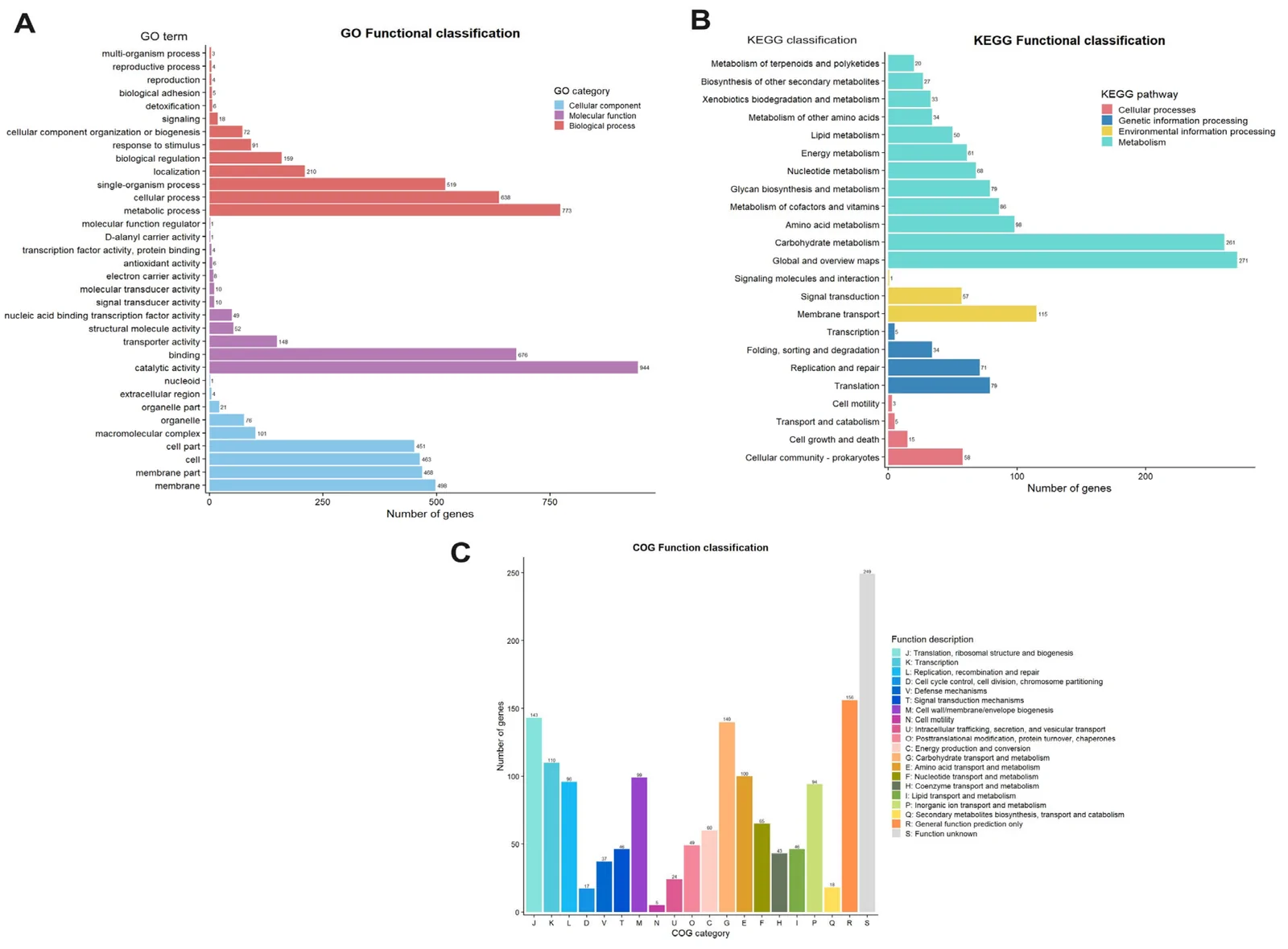
Functional annotation of *L. garvieae* AAHM-LG2501 and AAHM-LG2509. (**A**) GO functional classification, (**B**) KEGG functional classification, (**C**) COG functional classification.

GO functional annotation identified a total of 1537 genes associated with cellular component, molecular function, and biological process (Figure 5A). It should be noted that some genes were assigned to multiple GO categories. Within the cellular component category, most genes were associated with membrane (498 genes), membrane part (468 genes), cell (463 genes), and cell part (451 genes). In the biological process category, metabolic process (773 genes) was the predominant functional group, followed by cellular process (638 genes) and single-organism process (519 genes). In the molecular function category, catalytic activity (944 genes) represented the dominant function followed by binding activity (676 genes).

KEGG functional annotation predicted that 1531 genes were distributed across four KEGG pathway categories, including metabolism (1088 genes), genetic information processing (189 genes), environmental information processing (173 genes), and cellular processes (81 genes) (Figure 5B, Supplementary Table S2). At the KEGG pathways level, the high abundant pathways included biosynthesis of cofactors (84 genes), ABC transporters (65 genes), ribosome (52 genes), biosynthesis of amino acids (49 genes), carbon metabolism (47 genes), two-component system (41 genes), purine metabolism (39 genes), quorum sensing (39 genes), glycolysis/gluconeogenesis (38 genes), and phosphotransferase system (38 genes).

COG classification was performed to classify the predicted protein-coding genes into functional categories. The results showed that 1597 genes were assigned into 20 functional categories (Figure 5C, Supplementary Tables S2 and S3). Apart from the “Function unknown” and “General function prediction only” categories, the five most abundant functional groups were translation, ribosomal structure and biogenesis (143 genes), carbohydrate transport and metabolism (140 genes), transcription (110 genes), amino acid transport and metabolism (100 genes), and cell wall/membrane/envelope biogenesis (99 genes).

**Figure 6:**
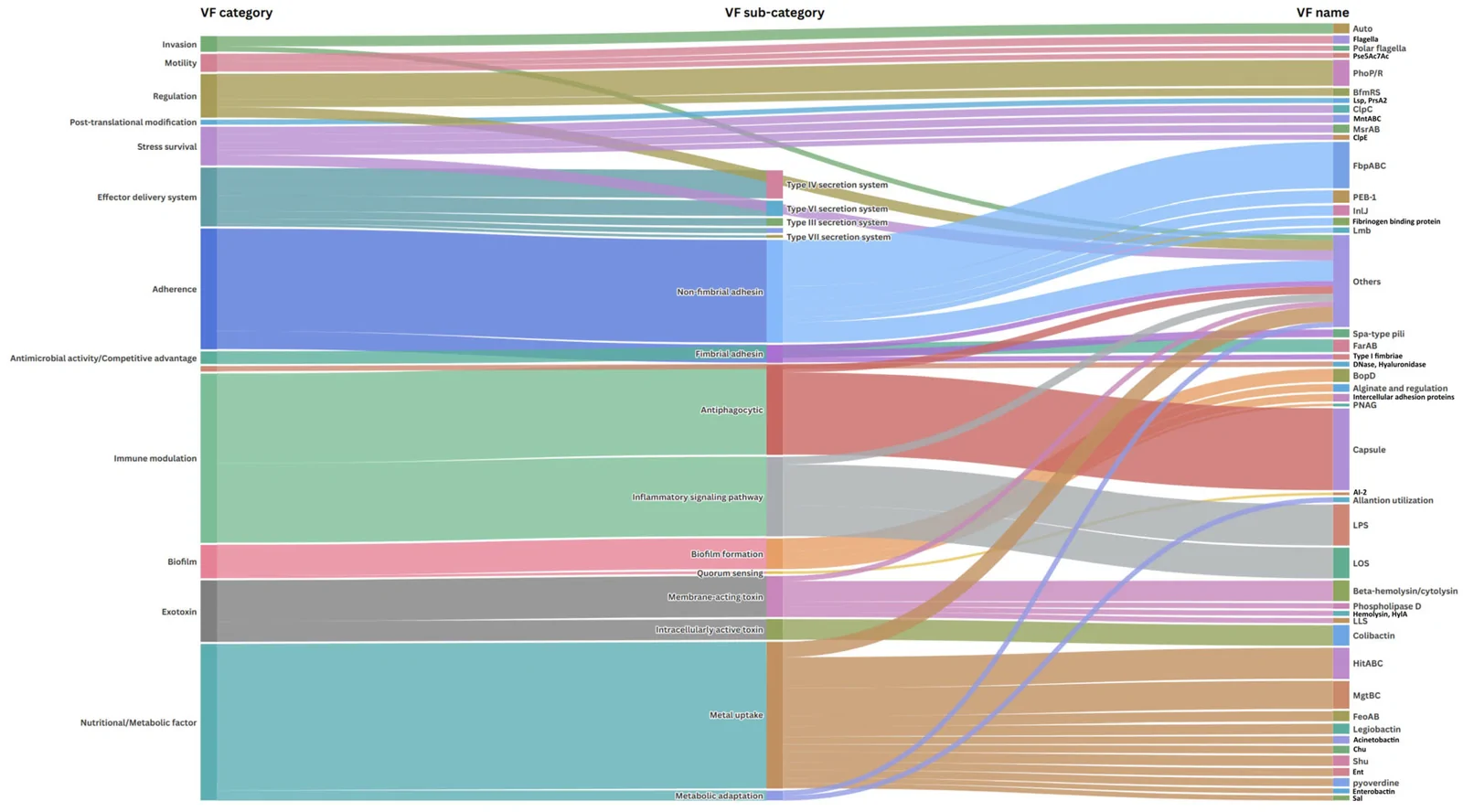
Sankey diagram showing the distribution of predicted virulence-associated genes of *L. garvieae* AAHM-LG2501 and AAHM-LG2509 identified using the Virulence Factor Database (VFDB). Flows indicate the association between virulence factor (VF) categories, VF sub-categories, and virulence genes. Node width reflects the relative abundance of genes within each VF category.

Virulence-associated gene prediction of *L. garvieae* AAHM-LG2501 and AAHM-LG2509 against VFDB identified a total of 288 virulence-associated genes. These genes were classified into 14 virulence factor categories containing 97 virulence factors. Among the identified categories, genes associated with immune modulation (66 genes) represented the largest proportion followed by nutritional/metabolic (61 genes), adherence (47 genes), exotoxins (24 genes), effector delivery systems (23 genes), regulation (17 genes), biofilm (13 genes), stress survival (15 genes), motility (7 genes), invasion (6 genes), antimicrobial activity/competitive advantage (5 genes), exoenzyme (2 genes), and post-translational modification (2 genes) (Figure 6, Supplementary Tables S2 and S3). Among the identified virulence factors, capsule-associated genes accounted for a notably high proportion (32 genes) compared with other individual virulence factors.

### 2.5. Antimicrobial Susceptibility and Antibiotic Resistance Properties

The antimicrobial susceptibility profiles of the *Lactococcus garvieae* isolates were determined using the disk diffusion method against 12 antimicrobial agents, including oxolinic acid (OA, 2 µg), nalidixic acid (NA, 30 µg), enrofloxacin (ENR, 5 µg), sulfamethoxazole-trimethoprim (SXT, 25 µg), tetracycline (TE, 30 µg), oxytetracycline (OT, 30 µg), doxycycline (DO, 30 µg), penicillin G (P, 10 µg), oxacillin (OX, 1 µg), amoxycillin (AML, 10 µg), ampicillin (AMP, 10 µg), and florfenicol (FFC, 30 µg) (Table 4). Notably, no inhibition zones were observed for NA, OA, and OX, indicating resistance of the isolate to these antimicrobial agents. Because interpretive criteria or clinical breakpoints for *L. garvieae* are currently unavailable, the antimicrobial susceptibility results were interpreted based on the relative diameters of inhibition zones. The isolate exhibited comparatively large inhibition zones against AML, AMP, P, TE, DO, OT, and FFC indicating higher susceptibility to these antimicrobial agents. In contrast, reduced inhibition zones were observed for ENR and SXT, suggesting decreased susceptibility.

The same number of AMR genes were detected from both *L. garvieae* AAHM-LG2501 and AAHM-LG2509 genomes. A total of 171 ARGs belonging to 29 antimicrobial classes and 117 ARG families were predicted using CARD with strict (3 genes) and loose hits (168 genes) (Supplementary Tables S4 and S5). Among these, genes annotated as being associated with resistance to multiple antimicrobial classes were the most abundant, accounting for 46 genes. Classification of AMR genes according to the antimicrobial classes to which they associated with resistance revealed that macrolide resistance genes were the most prevalent (29 genes), followed by fluoroquinolone (25 genes), peptide antibiotic (18 genes), aminoglycoside (14 genes), tetracycline (12 genes), and glycopeptide (12 genes). Because some ARGs are associated with resistance to multiple antimicrobial classes, individual genes may be represented in more than one resistance category. The ARGs identified as strict hits included *lsaD*, *vanT*, and *vanY*. It should be noted that several mutations were predicted to contribute to antimicrobial resistance through drug target alteration mechanisms. These variants were associated with resistance to multiple antibiotic classes such as fluoroquinolone, cephalosporin, aminoglycoside, etc. Notably, resistance-associated variants were identified in *gyrA* and *gyrB*, which encode recognized targets of fluoroquinolone antibiotics. Moreover, antimicrobial resistance phenotype prediction using ResFinder identified an acquired resistance gene, *mdtA* (identity 89.11%), in *L. garvieae* AAHM-LG2501 and AAHM-LG2509 genomes. Based on the ResFinder database, *mdtA* was predicted to confer resistance to macrolides and tetracyclines, including erythromycin, azithromycin, and tetracycline.

## 3. Discussion

The present study provides the first genome characterization of highly virulent *L. garvieae* isolates associated with a disease outbreak in cage-cultured Nile tilapia in Thailand. The two *L. garvieae* isolates were recovered from a disease outbreak in cage-cultured Nile tilapia in Ubon Ratchathani province, Thailand. Both isolates were confirmed as *L. garvieae* through a combination of conventional biochemical characterization, *16S rRNA* gene sequencing, and whole-genome sequencing. Notably, both isolates were assigned to MLST sequence type 95 (ST95), representing the first report of ST95 in Thailand. The isolates were also assigned to serotype I, and their genomes were further characterized for predicted virulence-associated genes and antimicrobial resistance determinants.

Most biochemical characteristics observed in the present study were consistent with those reported previously for *L. garvieae*. However, variability was observed in several carbohydrate fermentation tests, particularly for sucrose, lactose, mannitol, and sorbitol. Among these, sucrose fermentation has been proposed as a useful phenotypic marker for differentiating *L. petauri* from *L. garvieae* and *L. formosensis* when used in combination with MALDI-TOF MS or *16S rRNA* gene sequencing, as *L. petauri* is typically positive for sucrose utilization whereas *L. garvieae* and *L. formosensis* are negative [1]. In contrast, the isolates characterized in this study exhibited positive sucrose fermentation, consistent with previous reports that documented intraspecies variation and positive results in the sucrose test among *L. garvieae* isolates [6,7,18,19]. The variability observed in carbohydrate utilization patterns suggests that these phenotypic traits may not be universally conserved within *L. garvieae*. In addition, differences in biochemical testing methods among studies may have contributed to the inconsistent results and some studies may have relied on species identification methods with lower taxonomic resolution, so the possibility of species misidentification cannot be excluded.

Whole-genome sequencing analysis further demonstrated its value as a reliable tool for the taxonomic classification and species identification of lactococcosis-causing bacteria (LCB) in fish. The genome size of the *Lactococcus garvieae* isolates characterized in this study was consistent with that reported for other *L. garvieae* strains, which typically range from 1.9 to 2.0 Mb. Although plasmids have been identified in some *L. garvieae* isolates and have been implicated in the dissemination of virulence- and antimicrobial resistance-associated genes, no plasmids were detected in the present isolates. Based on in silico serotyping using serotype-specific primers, both isolates were assigned to serotype I according to the original serotype classification scheme of *L. garvieae*. Interestingly, MLST classified the isolates as ST95, which is the same sequence type as *L. garvieae* MS210922A isolated from farmed greater amberjack (*Seriola dumerili*) in Japan. This strain was subsequently proposed as a representative of the newly emerged serotype III [20]. The epidemiological study on *L. garvieae* serotype III reported more than 150 isolates recovered from striped jack (*Pseudocaranx dentex*) and greater amberjack in Japan during 2021 to 2023 [21]. Although ST95 has previously been associated with serotype III, the isolates in the present study were assigned to serotype I; the available data are insufficient to establish a specific association between ST95 and serotype III as it is unclear whether all serotype III isolates reported in the Japanese study were subjected to MLST. The different discriminatory properties of serotyping and MLST should be considered when selecting a typing approach according to the research objective. Serotyping is based on antigenic properties determined by slide agglutination and therefore provides information on antigenic variation among isolates, which may be relevant to studies of antigenic diversity and vaccine development. In contrast, MLST is based on sequence variation in selected housekeeping genes and is primarily useful for characterizing genetic lineages, population structure, epidemiological relationships, and evolutionary patterns.

In contrast to recent reports describing the distribution of LCB in Nile and red tilapia, in which *L. petauri* was identified more frequently than *L. garvieae*, all isolates recovered from the outbreak investigated in the present study were confirmed as *L. garvieae* [4,8]. A similar observation was reported by [10], where all isolates obtained from diseased red tilapia were identified as *L. garvieae*. However, species identification in that study was based primarily on *16S rRNA* gene sequencing, and phylogenetic analysis revealed overlap between *L. garvieae* and *L. petauri* clades, indicating limitations in species discrimination. Therefore, additional investigations involving a larger number of isolates from diverse outbreaks are required to better understand the epidemiology and host association of *L. garvieae* and *L. petauri* in tilapia.

The *L. garvieae* isolates characterized in this study were previously demonstrated to be highly pathogenic to Nile tilapia, causing systemic infection and mortality with an LD_50_ of 10^5^ CFU/fish following intraperitoneal injection and an LC_50_ of 10^6^ CFU/fish following immersion challenge [2]. Compared with the *L. garvieae* isolate reported by [8] which caused a cumulative mortality of 14.82% in Nile tilapia at an infective dose of 10^7^ CFU/fish, the isolates examined in the present study exhibited a higher degree of virulence. The experimental challenge results that demonstrated the high pathogenicity of these isolates provide an important context for interpreting the predicted virulence-associated repertoire. In particular, the extensive capsule-associated gene repertoire and the presence of hemolysin-associated genes may contribute to the ability of these isolates to evade host defenses, colonize host tissues, and cause systemic disease. Similar virulence determinants have been reported in other Gram-positive fish pathogens, including *Enterococcus faecalis*, *Streptococcus agalactiae*, and *Streptococcus iniae*, in which capsule production, adhesion, iron acquisition, and hemolytic activity contribute to host colonization and disease progression [22,23,24]. Although the present genomic analysis cannot establish a causal relationship between individual genes and the observed LD50/LC50 values, these findings provide candidate determinants that warrant further functional investigation.

The *cps* operon encoding the polysaccharide capsule was composed of ten *cps* genes (*cpsABCFGKO*, *cps4A*, *cps4I*) that were identified in our *L. garvieae* isolates. Variations within the capsule-associated gene cluster (CGC) were found between this study and [12] which reported that the virulent *L. garvieae* strain only carried *cpsLW* but possessed many *eps* genes (*epsRXABCD*) involved in exopolysaccharide (EPS) biosynthesis. The isolates characterized in the present study carried a broader repertoire of *cps* genes and showed high sequence similarity to capsule-associated genes reported in *E. faecalis* and *Streptococcus* spp. These findings suggest that variation exists in the genetic composition of the CGC among virulent *L. garvieae* strains. Nevertheless, the predominance of capsule-related genes in the present study, together with previous reports demonstrating the importance of capsule formation in virulence, supports the hypothesis that capsule production plays a significant role in the pathogenicity of *L. garvieae* by facilitating bacterial survival through evasion or interference with host immune responses and reduced susceptibility to phagocytosis.

In most studies, the predominant phenotype for virulent *Lactococcus* species is hemolysis which is commonly observed in most isolates. Hemolysins and cytolysins are critical to the pathogenesis of hyperacute hemorrhagic multi-organ septicemia in both *Lactococcus* spp. and *Streptococcus* spp. [14,22,23]. In the present study, four hemolysin/cytolysin-associated genes, including *hlyA* (hemolysin A), hemolysin III, *cylI* (3-ketoacyl-ACP synthase CylI), and *cylG* (3-ketoacyl-ACP reductase CylG), were identified in all *Lactococcus garvieae* isolates. Among these, hemolysin A and hemolysin III have previously been reported in both *L. garvieae* and *L. petauri* [5,11,18]. However, variation in hemolysin-associated gene profiles was observed among studies. In contrast to the findings of [5], which reported the presence of *tlyA* and *corC/hlyC*, the isolates characterized in the present study harbored *cylI* and *cylG*, which showed high sequence similarity to homologous genes described in *S. agalactiae* according to the VFDB. These findings suggest that diversity exists in the repertoire of hemolysin/cytolysin-associated genes among *L. garvieae* isolates. These exotoxins often co-occur with sortase-anchored proteins (LPxTG) and iron-uptake systems for survival in nutrient-poor host environments [11,15]. Eight genes encoding LPxTG cell wall anchor domain-containing proteins were identified in the *L. garvieae* isolates in this study. Among these, seven genes showed the highest sequence similarity to LPxTG proteins previously reported in *L. garvieae*, whereas one gene was most closely related to a homolog identified in *Bacillus cereus*. LPxTG-containing proteins are surface-associated adhesins that facilitate bacterial attachment to host tissues and have been recognized as important virulence determinants in *Lactococcus* spp. [11,12,14,15]. However, the LPxTG proteins identified in the present study were annotated based on sequence homology and were not further classified into the LPxTG1–7. Therefore, direct comparison with LPxTG protein and virulence in previous studies was not carried out.

The genes associated with the iron uptake system were also predominant across our *L. garvieae* isolates. A high number of these genes are associated with HitABC and MgtBC. These genes are related to nutritional/metabolic factors which contribute to bacterial adaptation and persistence within the host environment. The metal transport system plays an important role in metal homeostasis in *Streptococcus* species. HitABC is an ATP-binding cassette iron uptake system that supports bacterial survival in iron-limited host niches. A HitA lipoprotein was identified among surface-exposed proteins of *Streptococcus pyogenes* during pharyngitis and reacted with pooled human immune globulin, linking it to host interaction and in vivo fitness [25]. MgtBC is a highly conserved virulence factor found in several major intracellular pathogens such as *Mycobacterium tuberculosis* and *Salmonella enterica*, where it helps bacteria to survive within host macrophages [26,27,28]. MgtBC is a membrane-associated protein complex that has been proposed to function as a P-type ATPase involved in Mg^2+^ transport and other cation transport processes [28]. Beyond its role in ion homeostasis, MgtC has also been implicated in bacterial virulence. A study of *Salmonella* virulence [27] demonstrated that MgtC activates the PhoR histidine kinase, thereby inducing the Pho regulon and enhancing phosphate uptake which is required for normal *Salmonella* pathogenesis. Increased phosphate acquisition was associated with hypervirulence in *Salmonella* and a reduction in the non-replicating bacterial population within macrophages [27]. This observation is consistent with the results of the present study, in which PhoP/PhoR constituted one of the dominant virulence factors within the regulation category. The co-occurrence of MgtBC and the PhoP/PhoR regulatory system may indicate the importance of phosphate sensing and acquisition in the pathogenicity and host adaptation of *L. garvieae*.

Interpretation of the antimicrobial susceptibility results in *Lactococcus* spp. remains challenging because no species-specific clinical breakpoints are currently available, specifically bacteria isolated from aquatic animals. Consequently, some previous studies have applied interpretive criteria developed for other bacterial groups, including viridans group streptococci, enterococci, or staphylococci, or have adopted criteria from guidelines established for clinical isolates of human or terrestrial veterinary origin [4,6,14,15]. The use of breakpoints derived from different bacterial species and sources may introduce inconsistencies in the interpretation of inhibition zone diameters and complicate comparisons among studies. Therefore, in the present study, antimicrobial susceptibility results were primarily presented as inhibition-zone diameters rather than assigning categorical susceptible, intermediate, or resistant classifications based on potentially non-equivalent breakpoints. Establishment of standardized, species-appropriate susceptibility criteria based on a sufficiently large collection of *Lactococcus* isolates would improve the reliability and comparability of antimicrobial resistance surveillance in this genus. The establishment of standardized susceptibility criteria based on a large collection of *Lactococcus* isolates would therefore improve the consistency and comparability of antimicrobial resistance surveillance.

Resistance to first-generation quinolones including NA and OA was observed in all isolates in this study while ENR, the second generation, showed a quite low inhibition zone. Although we did not assign susceptibility interpretations to the ENR results because appropriate species- and aquatic-animal-specific breakpoints are unavailable, the inhibition-zone diameters observed in the present study would fall within the resistant criteria when compared with the interpretive criteria applied in a previous study [16,29]. This resistance phenotype corresponded to AMR gene profiles, which genes associated with fluoroquinolone resistance were detected. Notably, protein variants predicted to confer resistance to fluoroquinolone were detected in *gyrA* and *gyrB*. This mechanism plays an important role in fluoroquinolone resistance by altering the drug target. Substitutions in fluoroquinolone-targeted genes, *gyrA* (S83R) and *parC* (S80R), were reported in *L. petauri* which were associated with a levofloxacin-resistant phenotype [15]. From several studies, *lsaD*, *vanT*, and *vanY* were commonly detected in *Lactococcus* species and related to phenotype resistance to clindamycin and lincosamide. These genes were found in all isolates in this study which supports the idea of *lsaD* conferring the intrinsic clindamycin resistance of *L. garvieae*, and *vanT* and *vanY* were related to vancomycin resistance [1,5,11,14,15]. However, antimicrobial susceptibility testing against clindamycin, lincosamides, or vancomycin was not performed in the present study; therefore, the phenotypic relevance of these genes could not be confirmed. Tetracycline resistance in *Lactococcus* spp. has emerged as a concern because transferable plasmids carrying resistance genes such as *tetL* and *tetS* have been reported [1,11,14,15,17]. Although no plasmids were identified in the present isolates, several tetracycline resistance genes, including *tetT*, *tetO*, *tet38*, *tet42*, *tetA(58)*, and *tetA(60)*, were detected. Despite the presence of these genes, all isolates remained susceptible to the tested tetracycline antibiotics, suggesting that these low-confidence or potentially nonspecific chromosomally encoded determinants may have limited contributions to the observed phenotype, compared with plasmid-mediated tetracycline resistance mechanisms.

An important limitation of the present study is the restricted number and epidemiological origin of the sequenced isolates. Although 16 *L. garvieae* isolates were recovered during the outbreak investigation, whole-genome sequencing was performed for only two isolates, AAHM-LG2501 and AAHM-LG2509, originating from blood and gill samples, respectively. Moreover, both isolates were recovered within the same outbreak setting and showed highly similar genomic characteristics, including assignment to ST95 and serotype I and comparable virulence- and antimicrobial resistance-associated profiles. Thus, the two genomes should be regarded as representatives of a closely related outbreak-associated lineage rather than independent representatives of the broader *L. garvieae* population infecting tilapia in Thailand. The present data therefore support characterization of this particular outbreak-associated genotype but do not allow conclusions regarding the prevalence of ST95, genomic diversity, population structure, or geographical dissemination of *L. garvieae* at the national level. Expanded genomic surveillance incorporating multiple isolates from independent outbreaks, geographical regions, production systems, and sampling periods will be required to determine whether this lineage is locally restricted or more widely distributed in Thai tilapia aquaculture.

## 4. Materials and Methods

### 4.1. Field Case Identification and Outbreak Presentation

Two *Lactococcus garvieae* isolates, namely AAHM-LG2501 and AAHM-LG2509, were isolated from Nile tilapia during a lactococcosis outbreak from May to October 2025 in Ubon Ratchathani province, Thailand. Details of disease investigation and sample collection were provided in our previous study [2]. Briefly, fish with typical signs of lactococcosis, including hemorrhages on external organs, exophthalmia, and erratic swimming, were collected for bacterial isolation. A total of 16 isolates were obtained from blood and gill samples and were previously identified as *L. garvieae* based on near-full-length *16S rRNA* gene sequencing. For the present genome-level characterization, two isolates, AAHM-LG2501 and AAHM-LG2509, recovered from blood and gill samples, respectively, were selected for whole-genome sequencing as representatives of the outbreak-associated isolates obtained from the two sampled tissues. These isolates originated from the same outbreak investigation and were not selected to represent the broader genetic diversity of *L. garvieae* populations in Thailand. Accordingly, the genomic analyses in the present study were intended to characterize the outbreak-associated isolates rather than to infer population-level diversity or geographical distribution.

### 4.2. Bacterial Culture and Biochemical Tests

The bacteria were cultured on blood agar (tryptic soy agar (TSA) + 5% sheep blood) and incubated at 28 ± 2 °C for 24 h. Colony morphology, Gram staining and hemolysis activity were observed. Biochemical tests including catalase, oxidase, motile, indole, methyl red and Voges–Proskauer (MR-VP), citrate, urease, carbohydrate fermentation tests (glucose, lactose, sucrose, mannitol, sorbitol), and amino acid decarboxylase tests (ornithine, lysine, arginine) were performed under incubation at 28 ± 2 °C for 24 h. Capsule staining was carried out using nigrosin dye and observed under a light microscope.

### 4.3. DNA Extraction and Whole-Genome Sequencing

*L. garvieae* AAHM-LG2501 and AAHM-LG2509 were cultured in tryptic soy broth (TSB) (Difco^TM^, San Diego, CA, USA) and incubated at 28 ± 2 °C for 24 h. Genomic DNA was extracted from pure culture using a ZymoBIOMICS^TM^ DNA Miniprep Kit (Zymo Research Corporation, Irvine, CA, USA), following the manufacturer’s instructions. DNA purity and quantity were measured using a NanoDrop^TM^ spectrophotometer (Thermo Fisher Scientific, Waltham, MA, USA) prior to use for whole-genome sequencing. DNA libraries were prepared using the Nextera XT kit and sequencing was performed using an Illumina HiSeq platform with paired-end mode (Beijing Biomarker Technologies Co., Ltd., Beijing, China).

### 4.4. Whole-Genome Sequence Analysis

Quality trimming of raw sequence reads was performed using Trimmomatic v0.39 to exclude low-quality reads (Q < 20) and adapter sequences [30]. The filtered reads were assembled into draft genomes by Spades v3.6.2 [31]. Genome completeness and contamination assessment were determined with checkM [32]. Genome annotation and coding sequence (CDS) prediction were conducted using Prodigal v2.6.3 [33]. The predicted proteins were blasted against NCBI nr (non-redundant) (e-value cut off < 1 × 10^−5^) for species identification and genome annotation. Additionally, several databases were used for functional annotation including GO (gene ontology; accessed in December 2025), KEGG (Kyoto Encyclopedia of Genes and Genomes; accessed in December 2025), and eggNOG v6.0 (evolutionary genealogy of genes: Non-supervised Orthologous Groups; accessed in December 2025) [30,34,35,36,37]. The CRISPR system was analyzed using CRISPR Recognition Tool (CRT) v1.2 [38]. Plasmid sequences were screened using MOB-suite v3.1.9 [39].

### 4.5. Genome-Based Taxonomic Analysis

The Type Strain Genome Server (TYGS) was used to identify closely related type strains based on the Genome Blast Distance Phylogeny approach (GBDP) and to calculate digital DNA–DNA hybridization (dDDH) with the Genome-to-Genome Distance Calculator (GGDC) [40,41]. Average nucleotide identity (ANI) between genomes of this study and other *Lactococcus* spp. was calculated using pairwise comparison with MUMmer on the JSpeciesWS server [42]. Additionally, the multi-locus sequence typing (MLST) allelic profile was determined using PubMLST (Public databases for molecular typing and microbial genome diversity; accessed in March 2026). Additionally, in silico serotyping was carried out using the NCBI Primer-BLAST tool (https://blast.ncbi.nlm.nih.gov/Blast.cgi, accessed on 31 March 2026) with serotype-specific PCR primers (LGD-F and CGD-R) [43].

### 4.6. Antibiotic Resistance and Virulence Factors

Antibiotic resistance genes (ARGs) were identified using the online RGI (Resistance Gene Identifier) on CARD (Comprehensive Antibiotic Resistance Database) v4.0.1 with blastp (select criteria: perfect, strict, and loose hits; accessed in March 2026) [44]. ResFinder v4.7.2 was used to identify acquired genes and chromosomal mutations mediating antimicrobial resistance (identity cut off > 80%; accessed in March 2026) [45]. Virulence genes were identified based on the Virulence Factor Database (VFDB) using blastp against the full dataset of protein sequences (e-value cut off < 1 × 10^−5^; accessed in March 2026) [46].

### 4.7. Antimicrobial Susceptibility Test

Antimicrobial susceptibility testing was performed using the disk diffusion method. *L. garvieae* was cultured on TSA + 5% sheep blood and incubated at 28 °C for 24 h. Subsequently, a single colony was subcultured into TSB and incubated at 28 °C for 24 h with constant shaking. The bacteria were adjusted equal to 0.5 McFarland standard before being streaked onto Mueller–Hinton agar + 5% sheep blood. Antibiotic disks containing oxolinic acid (OA, 2 µg), nalidixic acid (NA, 30 µg), enrofloxacin (ENR, 5 µg), sulfa-trimethoprim (SXT, 265 µg), tetracycline (TE, 30 µg), oxytetracycline (OT, 30 µg), doxycycline (DO, 30 µg), penicillin G (P, 10 µg), oxacillin (OX, 1 µg), amoxicillin (AML, 10 µg), ampicillin (AMP, 10 µg), and florfenicol (FFC, 30 µg) were placed onto the agar. The plates were incubated at 28 °C for 24 h and zones of inhibition were measured using a vernier caliper.

## 5. Conclusions

In conclusion, the present study characterized two *L. garvieae* isolates recovered from a disease outbreak in cage-cultured Nile tilapia in Thailand using phenotypic and whole-genome sequencing approaches. Genome-based analyses confirmed the isolates as *L. garvieae* and demonstrated the utility of whole-genome sequencing for species-level characterization of closely related lactococcosis-causing bacteria. The isolates were assigned to ST95 and serotype I and harbored a diverse repertoire of predicted virulence-associated genes. Antimicrobial susceptibility testing revealed resistance to nalidixic acid, oxolinic acid, and oxacillin, while genomic analysis identified predicted antimicrobial resistance determinants and resistance-associated protein variants, including those associated with fluoroquinolone resistance. These findings provide baseline genomic and phenotypic information on virulence-associated characteristics and antimicrobial resistance in outbreak-associated *L. garvieae* from Thai Nile tilapia. However, given the limited number of isolates and sampling from a single outbreak, these findings should not be interpreted as evidence of the epidemiological distribution, emergence of resistant *L. garvieae* populations, or regional transmission patterns. Accordingly, these findings provide baseline genomic and phenotypic information for this outbreak-associated ST95/serotype I lineage but do not establish its prevalence, population structure, or geographical distribution. Broader genomic surveillance involving isolates from independent outbreaks, geographical regions, and sampling periods is required to determine the diversity and dissemination of *L. garvieae* in Thai aquaculture.

## Figures and Tables

**Table 1:** Biochemical characteristics of *L. garvieae* AAHM-LG2501 and AAHM-LG2509.

Biochemical Test	Result
Catalase	Negative
Oxidase	Negative
Motile	Negative
Indole	Negative
MR	Negative
VP	Positive
Carbohydrate fermentation test	
Glucose	Positive
Lactose	Positive
Sucrose	Positive
Mannitol	Positive
Sorbitol	Positive
Decarboxylase test	
Ornithine	Negative
Lysine	Negative
Arginine	Positive

Both AAHM-LG2501 and AAHM-LG2509 showed identical biochemical profiles.

**Table 2:** Assembly statistics and genome features of *L. garvieae* AAHM-LG2501 and AAHM-LG2509.

	*L. garvieae* AAHM-LG2501	*L. garvieae* AAHM-LG2509
Contig number	14	13
Contig N50	275,740 bp	275,743 bp
Mean coverage	257.4×	298.7×
Completeness	100%	100%
Genome size	1,955,034 bp	1,954,741 bp
GC content	38.9%	38.9%
Number of CDS	1866	1870
Number of rRNA	7	7
Number of tRNA	50	50
Number of CRISPR	8	8
Total gene length	1,730,685 bp	1,731,282 bp
Genes assigned to NCBI nr	1845	1847
Genes assigned to GO	1537	1537
Genes assigned to eggNOG	1597	1597
Genes assigned to KEGG	1531	1531
Genes assigned to VFDB	288	288
Genes assigned to CARD	171	171
Accession number	JCAFHE000000000	JCAFHD000000000

**Table 3:** dDDH and G + C difference values between *L. garvieae* of this study and type strains.

Query Strain	Subject Strain	dDDH (d0, %)	C.I. (d0, %)	dDDH (d6, %)	C.I. (d6, %)	G + C Difference (%)
*L. garvieae* AAHM-LG2501	*L. garvieae* NBRC 100934	84.4	[80.6–87.5]	87.9	[84.9–90.3]	0.34
	*L. garvieae* DSM 20684	84.5	[80.8–87.7]	88	[85.0–90.4]	0.36
	*E. seriolicida* ATCC 49156	88.6	[85.2–91.3]	90.9	[88.3–93.0]	0.04
	*L. formosensis* subsp. *bovis* LMG 30663	65.8	[61.9–69.4]	61.5	[58.2–64.7]	1.33
	*L. formosensis* subsp. *bovis* DSM 100577	66.1	[62.2–69.7]	61.7	[58.4–64.9]	1.34
	*L. formosensis* NBRC109475	73	[69.0–76.7]	67.1	[63.7–70.3]	0.98
	*L. petauri* 159469	68.9	[65.0–72.6]	66.6	[63.2–69.8]	1.2
	*L. intestinalis* M2458	30.8	[27.4–34.4]	28.8	[25.9–31.9]	0.64
	*L. muris* DSM 109779T	32	[28.6–35.5]	29.7	[26.7–32.8]	0.3
	*L. ileimucosae* DSM 107391T	31.9	[28.5–35.5]	29.6	[26.6–32.7]	0.61
*L. garvieae* AAHM-LG2509	*L. garvieae* NBRC 100934	84.4	[80.6–87.5]	87.9	[84.9–90.3]	0.34
	*L. garvieae* DSM 20684	84.5	[80.8–87.7]	88	[85.0–90.4]	0.35
	*E. seriolicida* ATCC 49156	88.6	[85.2–91.3]	90.9	[88.3–93.0]	0.04
	*L. formosensis* subsp. *bovis* LMG 30663	65.8	[61.9–69.4]	61.5	[58.2–64.7]	1.33
	*L. formosensis* subsp. *bovis* DSM 100577	66.1	[62.2–69.7]	61.7	[58.4–64.9]	1.34
	*L. formosensis* NBRC109475	73	[69.1–76.7]	67.1	[63.7–70.3]	0.98
	*L. petauri* 159469	68.9	[65.0–72.6]	66.6	[63.2–69.8]	1.19
	*L. intestinalis* M2458	30.8	[27.4–34.4]	28.8	[25.9–31.9]	0.65
	*L. muris* DSM 109779T	32	[28.6–35.5]	29.7	[26.7–32.8]	0.3
	*L. ileimucosae* DSM 107391T	31.9	[28.5–35.5]	29.6	[26.6–32.7]	0.61

**Table 4:** Antimicrobial susceptibility profiles and antimicrobial resistance genes.

*L. garvieae* Isolates	AAHM-LG2501	AAHM-LG2509
Zone of inhibition (mm)		
OA (2 µg)	6 ± 0 (R)	6 ± 0 (R)
NA (30 µg)	6 ± 0 (R)	6 ± 0 (R)
ENR (5 µg)	16 ± 0.7	14 ± 0.7
SXT (25 µg)	18 ± 0	23 ± 0
AML (10 µg)	23 ± 0.7	25 ± 0.7
AMP (10 µg)	29 ± 0.7	30 ± 0.7
P (10 µg)	28 ± 0	25 ± 0
OX (1 µg)	6 ± 0 (R)	6 ± 0 (R)
TE (30 µg)	28 ± 0	26 ± 0
DO (30 µg)	26 ± 0	27 ± 0
OT (30 µg)	26 ± 0.7	29 ± 0.7
FFC (30 µg)	26 ± 0.7	25 ± 0.7
Antimicrobial resistance genes		
CARD	*lsaD*, *vanT*, *vanY*	*lsaD*, *vanT*, *vanY*
ResFinder	*mdtA*	*mdtA*

R = resistant.

## Data Availability

The near-full-length 16S rRNA gene sequences generated in this study have been deposited in the NCBI GenBank database under accession numbers PZ007933–PZ007948, corresponding to isolates AAHM-LG2501–AAHM-LG2516, respectively. The whole-genome sequences of isolates AAHM-LG2501 and AAHM-LG2509 have been deposited in GenBank under accession numbers JCAFHE000000000 and JCAFHD000000000, respectively, under BioProject PRJNA1455887. Other data supporting the findings of this study are available from the corresponding authors upon reasonable request.

## Supplementary Materials

The following supporting information can be downloaded at: https://www.mdpi.com/article/10.3390/ijms27177582/s1.
